# Impaired dual-task gait in Parkinson’s disease is associated with brain morphology changes

**DOI:** 10.1007/s00702-024-02758-2

**Published:** 2024-02-28

**Authors:** Radim Krupička, Christiane Malá, Slávka Neťuková, Tereza Hubená, Filip Havlík, Ondrej Bezdicek, Petr Dušek, Evžen Růžička

**Affiliations:** 1 Department of Biomedical Informatics, Faculty of Biomedical Engineering, Czech Technical University in Prague, Prague, Czech Republic; 2 Department of Neurology, Centre of Clinical Neuroscience, First Faculty of Medicine, Charles University and General University Hospital, Prague, Czech Republic

**Keywords:** Dual-task cost, Gait speed, Stride length, Stepping cadence, Timed up and go test, Morphometric correlation analysis

## Abstract

In Parkinson’s disease (PD), impaired gait and cognition affect daily activities, particularly in the more advanced stages of the disease. This study investigated the relationship between gait parameters, cognitive performance, and brain morphology in patients with early untreated PD. 64 drug-naive PD patients and 47 healthy controls (HC) participated in the study. Single- and dual-task gait (counting task) were examined using an expanded Timed Up & Go Test measured on a GaitRite walkway. Measurements included gait speed, stride length, and cadence. A brain morphometry analysis was performed on T1-weighted magnetic resonance (MR) images. In PD patients compared to HC, gait analysis revealed reduced speed (*p* < 0.001) and stride length (*p* < 0.001) in single-task gait, as well as greater dual-task cost (DTC) for speed (*p* = 0.007), stride length (*p* = 0.014) and cadence (*p* = 0.029). Based on the DTC measures in HC, PD patients were further divided into two subgroups with normal DTC (PD-nDTC) and abnormally increased DTC (PD-iDTC). For PD-nDTC, voxel-based morphometric correlation analysis revealed a positive correlation between a cluster in the left primary motor cortex and stride-length DTC (*r* = 0.57, *p* = 0.027). For PD-iDTC, a negative correlation was found between a cluster in the right lingual gyrus and the DTC for gait cadence (*r*=-0.35, p_FWE_ = 0.018). No significant correlations were found in HC. The associations found between brain morphometry and gait performance with a concurrent cognitive task may represent the substrate for gait and cognitive impairment occurring since the early stages of PD.

**Supplementary Information:**

The online version contains supplementary material available at 10.1007/s00702-024-02758-2.

## Introduction

Impairments of gait and cognition typically emerge in advanced Parkinson’s disease (PD) and progressively affect patients’ daily living activities. A significant impairment of gait parameters can already be present in the early stages of PD (Zhang et al. 2022). A meta-analysis by Zanardi et al. (Zanardi et al. 2021) included 72 studies demonstrating clear differences in gait speed, cadence and stride length between PD and healthy controls (HC).

Several studies have already shown a relationship between cognitive performance and gait parameters of PD patients (Amboni et al. 2012; Varalta et al. 2015) suggesting specific associations of impairments in both domains. By adding a cognitive activity alongside the walking task, one can assess Dual Task performance, enabling the calculation of Dual Task Cost (DTC). This metric provides insights into the extent of deterioration resulting from the added cognitive load while walking. It was shown that DTC of gait parameters including stride length, cadence and gait speed differed significantly between PD and HC and that DTC are related to cognitive performance measures in patients with PD (Harrie et al. 2022). Moreover, a recent study (Johansson et al. 2021) showed differences in dual-task gait prioritization strategy (cognitive/walking) between PD patients with and without mild cognitive impairment.

While morphological changes in the brains of PD patients affected by gait disturbances are relatively well explored, the contribution of associated cognitive impairments is less clear and has recently been a topic of research. Functional magnetic resonance imaging (FMRI) during performance of a cognitive and motor dual-task showed differences in brain activation between PD patients and HC (Nieuwhof et al. 2017). Using functional near-infrared spectroscopy in PD patients during walking, enhanced activation of the premotor cortex and supplementary motor area was found when performing a cognitive dual-task, representing a possible coping strategy for the additional cognitive load (Liu et al. 2022).

Although several studies have investigated brain activation during dual-tasking (Nieuwhof et al. 2017; Hirata et al. 2020), to our knowledge, no study in PD patients addressed possible associations between morphological changes in the brain and varying degrees of cognitive task interference expressed as DTC. This study aims to answer the question of whether the severity of gait impairment during cognitive task execution correlates with brain morphology. This information would provide a better insight into the mechanisms underlying the interplay between cognitive performance and gait in PD patients.

## Methods

### Participants

We included 64 drug-naive PD patients (mean age 58.2 ± 12.3 years) and 47 healthy controls (HC) (mean age 60.4 ± 9.2 years) (Table 1). All participants completed at least 9 years of education. Patients with PD were diagnosed according to the Movement Disorder Society clinical diagnostic criteria for PD (Postuma et al. 2015) and investigated before the introduction of dopaminergic therapy. In all patients enrolled in this study, a clear beneficial response to dopaminergic therapy was observed at follow-up.

**Table 1:** Clinical characteristics of participants

	PD	Controls	*p*-value
Male sex	34/64 (53%)	29/47 (62%)	0.44
Age (years)	58.2 (12.3, 33–81)	60.4 (9.2, 43–75)	0.31
Symptom duration (years)	1.7 (1.3, 0.1–5.3)	n/a	n/a
MoCA	26.5 (1.75, 24–30)	26.5 (1.72, 24–30)	0.96
TMT-B	91 (40, 44–213)	80 (23.8, 42–149)	0.10
MDS - UPDRS III	28.0 (12.6, 6–70)	n/a	n/a

Note: Data are mean (SD, range) or number/sample size (%) including *p*-values analyzed using t-test test or Mann–Whitney U-test

Abbreviations: MDS-UPDRS, Movement Disorder Society Unified Parkinson’s Disease Rating Scale; MoCA, Montreal Cognitive Assessment; n/a, not applicable; PD, Parkinson disease; TMT-B, Trail Making Test, Part B

The healthy control group was acquired by advertising to the general public. The exclusion criteria for HC were neurological or psychiatric disorders, the use of psychoactive substances, concurrent oncological or other major somatic diseases, the presence of REM sleep behavioral disorder. For both groups, participants with major hearing and vision problems were excluded and only non-demented, cognitively normal participants with MoCA scores of 24 or higher were included. The cut-off value of 24 refers to the results of a normative study for the Czech population (Kopecek et al. 2017). All included participants had a full score of 3 points in MoCA Serial 7 Subtraction Task, i.e., they didn’t show any impairment in the cognitive test that was also used as a competitive task in dual-task walking.

All participants underwent a detailed medical interview, and a neurological and neuropsychological examination (the battery included five cognitive domains, as recommended by the Movement Disorder Society: attention and working memory; executive functions; language; delayed recall, visuospatial abilities (Litvan et al. 2012). The Movement Disorder Society Unified Parkinson’s Disease Rating Scale, motor subscale (MDS-UPDRS part III) (Goetz et al. 2008) and the Montreal Cognitive Assessment (MoCA) were used to assess motor and cognitive performance (Hobson 2015). All participants gave informed consent. The study received approval from the Ethics Committee of the General University Hospital in Prague and has been performed in accordance with the ethical standards established in the 1964 Declaration of Helsinki.

### Gait assessment

All participants completed the expanded Timed Up & Go Test (TUG) (Wall et al. 2000): get up from a chair, walk 10 m at the preferred walking speed, turn around, walk back and sit down again. TUG was performed twice. For data measurements, a 5.15 m long and 0.9 m wide pressure walkway (Platinum model GAITRite®, CIR System Inc.) was placed 2.43 m from the chair in the middle of the straight gait walkway. Participants were instructed to walk at a normal pace under two different settings: (i) in the single-task (ST) condition and (ii) in the dual-task (DT) condition while performing serial subtraction, counting down from 100 by sevens. During straight walk, individual gait cycles were detected and analysed, and the gait speed, stride length and cadence were investigated (Zampieri et al. 2010).

Dual-task cost (DTC) was calculated for all monitored gait parameters (speed, stride length, and cadence), to evaluate the relative impact of the additional cognitive task on gait performance:

$$DTC=\frac{DTvalue-STvalue}{STvalue}$$

The DTC parameter includes information about how the additional cognitive load in DT influences gait performance and thus combines gait and cognitive assessment which was further used to divide patients into subgroups. A principal component analysis (PCA) was performed that included all DTC gait parameters in the HC data set. Based on the gait evaluation and comparison with the control group, patients with PD were divided into two subgroups with normal (nDTC) and abnormal DTC (iDTC). The criterion for inclusion in the nDTC group was to have the DTC first PCA component above the 10th percentile of HC, while the iDTC group consisted of individuals with values below the 10th percentile.

### Image acquisition and pre-processing

A 3T MRI scanner (Siemens Skyra 3T, Siemens Healthcare, Erlangen, Germany) with a 32-channel head coil was used to perform the examination.

Morphometry analysis was performed on T1-weighted 3D magnetization-prepared rapid acquisition with gradient echo (MPRAGE) images in the axial plane with the following acquisition parameters: repetition time (TR), 2,200 ms; echo time (TE), 2.4 ms; inversion time (TI) 900 ms; flip angle (FA) 8°; field of view (FOV) 230 × 197 × 176 mm; spatial resolution 1 mm^3^ isotropic.

The pre-processing and segmentation of T1 weighted images were performed with the Computational Anatomy Toolbox software (CAT12), version 12.8.2 (Gaser et al. 2022) implemented in the statistical parametric mapping software (SPM, version 7771) (Friston 2007) in Matlab (The MathWorks Inc. 2022). By reviewing one slice of every brain, a visual quality check was performed to find obvious artefacts in the scans and inaccurately orientated images. The homogeneity of the data was checked by applying the CAT batch of data quality. The segmentation quality for every single image was accepted at a minimum of C + in all quality parameters. The modulated, normalized grey matter segments were smoothed using a Gaussian kernel with an 8 mm^3^ full width at half maximum to perform the voxel-based morphometry (VBM).

### Statistical analysis

Differences in gait parameters between groups were performed by using the general linear model with age and sex as covariates. VBM analysis was performed using a multiple regression model with the covariate DTC (speed or stride length or cadence), total intracranial volume (TIV), age, and sex. The statistical map for the correlation analysis was thresholded at cluster level at the statistical level *p* < 0.05 corrected by family-wise error (FWE).

The subgroup of HC-iDTC was excluded from all statistical analyses because of the low number of participants.

## Results

### Gait analysis

**Fig. 1:**
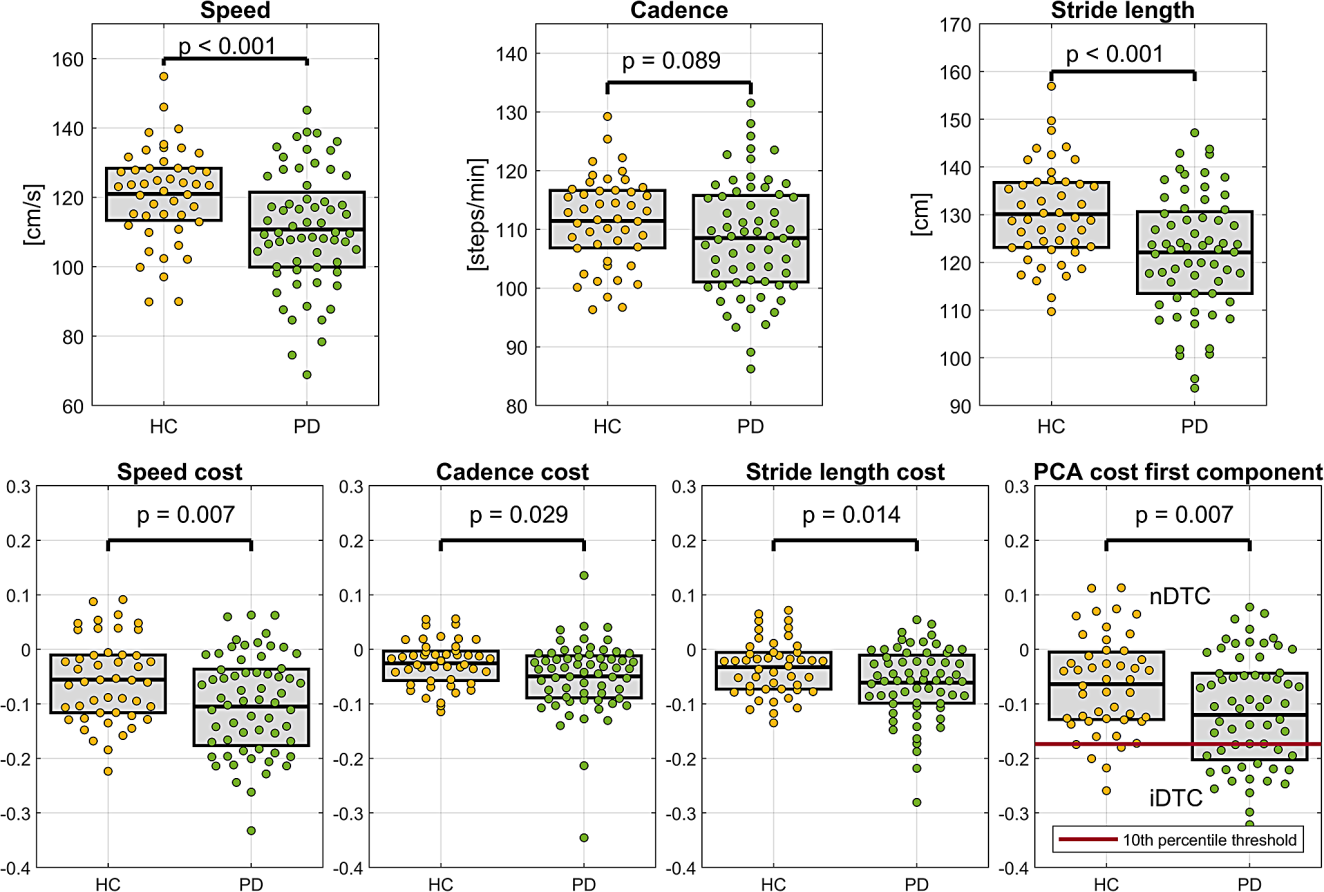
Differences in single- and dual-task gait between healthy controls (HC) and Parkinson’s disease patients (PD). The influence of age and gender was mitigated using a general linear model. The graph “PCA cost first component” defines nDTC and iDTC subgroups distinguished by 10th percentile of HC (red line)

In single-task gait, decreased speed (*p* < 0.001) and stride length (*p* = 0.001) were observed in PD patients compared to HC, with no significant difference in cadence. PD patients also showed greater DTC compared to HC across all parameters: speed (*p* = 0.007), stride length (*p* = 0.014) and cadence (*p* = 0.029) (Fig. 1).

The first PCA component derived from DTC parameters linearly combined gait speed cost (with a multiplication coefficient of 0.8), cadence cost (-0.01) and stride length cost (0.6) and differed between PD and HC (*p* = 0.007). The 10th percentile of the first PCA component in HC divided PD patients into a subgroup with normal DTC (PD-nDTC) (*n* = 44/ 25 males, mean age 58.6 ± 12.2 years, MoCA 26.6 (1.9, 24–30), MDS-UPDRS III 24.3 (9.7, 6–43) ) and a subgroup with abnormally increased DTC (PD-iDTC) (*n* = 20 / 11 males, mean age 57.3 ± 13.0 years, MoCA 26.4 (1.4, 24–29), MDS-UPDRS III 36.2 (14.5, 14–70)) (see Fig. 1).

Single-task gait parameters showed no differences between the PD-nDTC and PD-iDTC subgroups.

### VBM analysis

A group comparison between PD-nDTC and PD-iDTC did not reveal any significant differences after a FWE correction. Without the FWE correction, a cluster in the left frontal inferior lobe pars triangularis had a significantly higher grey matter density in PD-nDTC than in PD-iDTC (p_uncorr_ = 0.013).

**Fig. 2:**
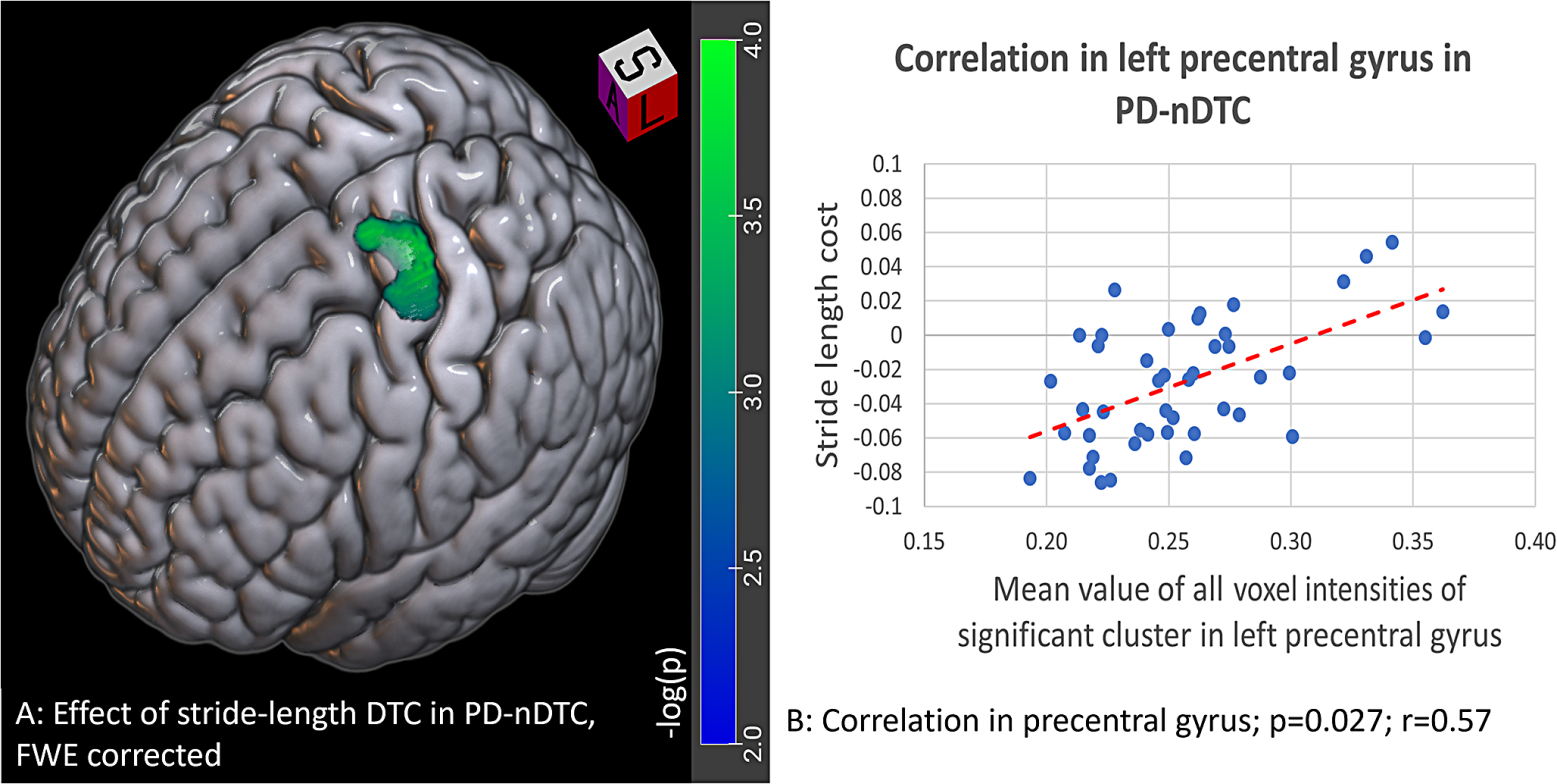
Results of the correlation analysis of brain morphometry with DTC of gait parameters for Parkinson’s disease patients without gait-cognitive impairment (PD-nDTC). The color scale represents the negative decimal logarithm of the *p*-value. **A**: Significant cluster in the left precentral gyrus for a positive correlation with stride length DTC in PD-nDTC, p_FWE_ = 0.027. **B**: Correlation of gray matter density in significant cluster in precentral gyrus with stride length DTC

For PD-nDTC, a cluster in the left primary motor cortex positively correlated with the stride-length DTC (*r* = 0.57, p_FWE_ = 0.03) (see Fig. 2). The corresponding cluster on the right hemisphere did not reach a significant level of p_uncorr_<0.05. A similarly located cluster correlated with speed cost (*r* = 0.51, p_uncorr_ = 0.02) but the correlation did not reach significant level after FWE correction. No significant correlations were found for cadence DTC in PD-nDTC.

**Fig. 3:**
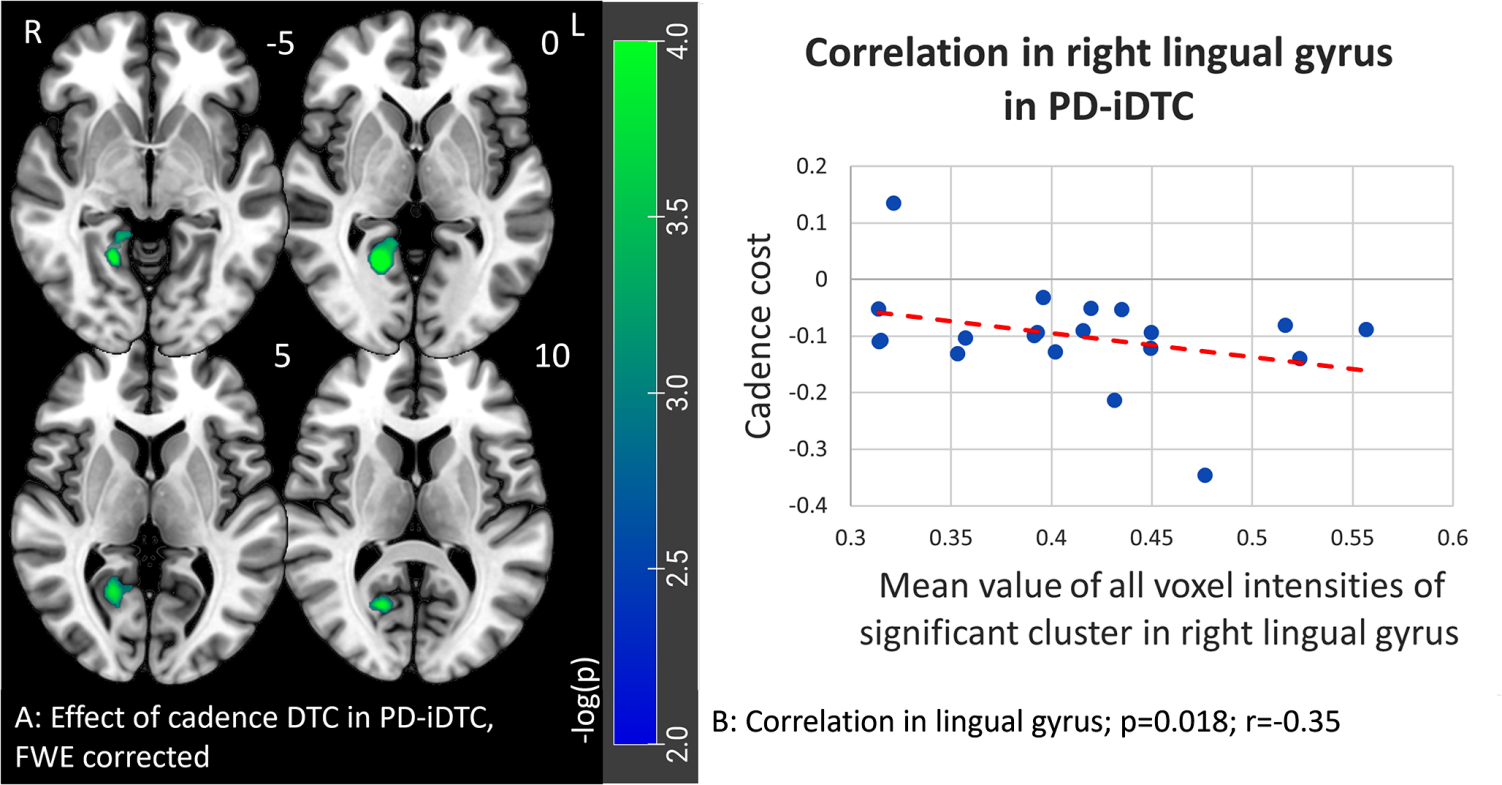
Results of the correlation analysis of brain morphometry with DTC of gait parameters for Parkinson’s disease patients with gait-cognitive impairment (PD-iDTC). The color scale represents the negative decimal logarithm of the *p*-value. **A**: Significant cluster in the right lingual gyrus for a negative correlation with cadence DTC in PD-iDTC, p_FWE_ = 0.018. **B**: Correlation of gray matter density in significant cluster in lingual gyrus with cadence DTC

In the PD-iDTC group, a negative correlation with cadence DTC was found for a cluster in the right lingual gyrus (*r* = -0.35, p_FWE_ = 0.02) (see Fig. 3). Speed DTC showed a correlation in a similar location with a level under the border of significance (*r* = -0.26, p_uncorr_ = 0.06). For stride length cost, no significant correlation could be detected in the PD-iDTC group. The correlations with the same cluster in the lingual gyrus could neither be found in the PD-nDTC subgroup.

No correlations with brain morphology were found in HC and in the whole PD group.

## Discussion

In summary, gait analysis in our group of patients with early untreated PD compared with HC showed reduced speed and stride length in single-task gait as well as greater dual-task cost in speed, stride length, and cadence. The results of single-task gait analysis are consistent with previous research in which patients with PD exhibited reduced walking speed associated with shorter stride length (Zanardi et al. 2021). In contrast to some previous studies, our cohort did not show increased gait cadence (Morris et al. 1998; Chee et al. 2009). This may be due to our TUG setting, where participants were not instructed to walk with the highest possible speed, thus it is unlikely that they tried to compensate for decreasing stride length with a higher cadence. Another important factor may be the short duration and early stage of the disease in the majority of our patients, whereby increases in cadence are associated with more advanced PD (Morris 1998) and with gait freezing (Chee 2009). Also, the observed effects of dual-tasking are consistent with previous research demonstrating greater DTC even in non-demented PD patients (Raffegeau et al. 2019).

The main contribution of the present study are the findings of morphometric correlation analysis suggesting that there are different brain regions involved in motor-cognitive dual-task performance in PD patients with normal DTC and with increased DTC values. This is reminiscent of the results of a recent study that found differences between cognitively impaired and unimpaired PD patients in focus priority during dual-task performance (Johansson et al. 2021). While cognitively impaired patients appeared to focus more on the cognitive task, patients without cognitive impairment focused more on the motor task (Johansson et al. 2021). Accordingly, in our PD-nDTC patients, the higher the gray matter density in the left precentral gyrus, i.e., the primary motor cortex, the more patients presumably tend to prioritize the motor task, as reflected by the normal DTC of stride length and speed. In contrast, in the PD-iDTC patients, voxel-wise analysis showed a negative correlation between cadence DTC and a cluster in the right lingual gyrus. With its involvement in spatial processing and visual imagery of numbers and letters, the lingual gyrus function can be involved in solving the “counting backwards” arithmetic task as part of dual-task performance (Bogousslavsky et al. 1987). If the stride cadence DTC was inversely correlated with gray matter density in the lingual gyrus, it is consistent with the previous observation that cognitively impaired patients concentrate more on the cognitive than on the motor task (Johansson et al. 2021). Of note, there was no statistical difference in TMT-B, a classical measure of executive dysfunction, between PD and HC. Our finding thus suggests a connection of increased gait cadence with specific cognitive defect associated with an incipient atrophy of the lingual gyrus without being driven by executive dysfunction. Importantly, atrophy of occipital cortex including lingual gyrus has been consistently reported in late-stage PD (Wilson et al. 2019) and associated with cognitive impairment (Burton 2004), hallucinations (Watanabe et al. 2013) and gait freezing (Tessitore et al. 2012).

The fact that none of these correlations could be found in the HC group implies that our observations in PD are related to neurodegenerative brain changes and/or compensatory mechanisms. The results presented here suggest different patterns of neurodegeneration and neuroplasticity in iDTC and nDTC patients, leading to the findings of different relationships between cortical gray matter intensity and DTC values for individual gait parameters. We speculate that nDTC patients may be able to take advantage of compensatory neuroplastic changes in the motor cortex to keep normal dual-task performance while in iDTC patients, the impaired performance in dual-task is directly related to the degree of lingual gyrus degeneration. Yet, neither the gray matter intensity in the lingual gyrus nor in the precentral gyrus differed between the two PD subgroups, leading to the assumption that the differences in correlating clusters are not influenced by a general atrophy of these regions in either subgroup. Thus, only a follow-up study assessing longitudinal evolution of brain morphometry along with dual-task gait performance can prove whether our theory is correct.

A limitation of the study is the lack of recording patient´s performance in the concurrent cognitive task. Another problem may be patients’ inconsistent motivation and variations in understanding the instructions. However, this is an inherent shortcoming of all gait studies, especially with dual-tasking (Nieuwhof et al. 2017). During further studies, it would be interesting to record the cognitive performance during dual-task gait as a separate parameter to investigate if the intergroup differences in prioritization and compensation of combined motor and cognitive load are also recognizable in the performance of the cognitive task. Intra-individual variability in gait during the assessment is also a potential source of bias. Although we attempted to proceed in a standard unchanging way for all participants, striving for a similar level of motivation, we cannot rule out some influence of intra-individual variability. However, this should not be larger than in other dual-task studies in PD patients using the same methodology (Johansson et al. 2021). Another limitation is the difference in sample size between the two subgroups, particularly the smaller sample size of the iDTC group, which carries the risk that the observed correlations are influenced by individual morphological features of the brains of patients in this group.

In conclusion, our study expands knowledge on the relationship between dual-task gait performance and brain morphological parameters in patients with early PD. When comparing the findings in patients with normal and abnormally elevated DTC, the correlations between gray matter intensity in different cortical clusters and DTC values for speed, stride length, and cadence appear to correspond to distinct mechanisms of motor or cognitive prioritization and compensation.

## Electronic Supplementary Material

Below is the link to the electronic supplementary material.

Supplementary Material 1

## References

[CR1] Amboni M, Barone P, Iuppariello L et al (2012) Gait patterns in parkinsonian patients with or without mild cognitive impairment. Mov Disord 27:1536–1543. 10.1002/mds.25165 23032876 10.1002/mds.25165

[CR2] Bogousslavsky J, Miklossy J, Deruaz JP et al (1987) Lingual and fusiform gyri in visual processing: a clinico-pathologic study of superior altitudinal hemianopia. J Neurol Neurosurg Psychiatry 50:607–614. 10.1136/jnnp.50.5.607 3585386 10.1136/jnnp.50.5.607 PMC1031973

[CR3] Burton EJ (2004) Cerebral atrophy in Parkinson’s disease with and without dementia: a comparison with Alzheimer’s disease, dementia with Lewy bodies and controls. Brain 127:791–800. 10.1093/brain/awh088 14749292 10.1093/brain/awh088

[CR4] Chee R, Murphy A, Danoudis M et al (2009) Gait freezing in Parkinson’s disease and the stride length sequence effect interaction. Brain 132:2151–2160. 10.1093/brain/awp053 19433440 10.1093/brain/awp053

[CR5] Friston KJ (2007) Statistical parametric mapping: the analysis of functional brain images, 1st edn. Elsevier / Academic, Amsterdam Boston

[CR6] Gaser C, Dahnke R, Thompson PM et al (2022) CAT – A Computational Anatomy Toolbox for the Analysis of Structural MRI Data. Neuroscience 10.1093/gigascience/giae049 PMC11299546 39102518

[CR7] Goetz CG, Tilley BC, Shaftman SR et al (2008) Movement Disorder Society-sponsored revision of the Unified Parkinson’s Disease Rating Scale (MDS‐UPDRS): Scale presentation and clinimetric testing results. Mov Disord 23:2129–2170. 10.1002/mds.22340 19025984 10.1002/mds.22340

[CR8] Harrie A, Hampstead BM, Lewis C et al (2022) Cognitive correlates of dual tasking costs on the timed up and go test in Parkinson disease. Clin Parkinsonism Relat Disorders 7:100158. 10.1016/j.prdoa.2022.100158 10.1016/j.prdoa.2022.100158 PMC9358459 35957864

[CR9] Hirata K, Hattori T, Kina S et al (2020) Striatal dopamine denervation impairs Gait Automaticity in Drug-Naïve Parkinson’s Disease patients. Mov Disord 35:1037–1045. 10.1002/mds.28024 32163636 10.1002/mds.28024

[CR10] Hobson J (2015) The Montreal Cognitive Assessment (MoCA). OCCMED 65:764–765. 10.1093/occmed/kqv078 10.1093/occmed/kqv078 26644445

[CR11] Johansson H, Ekman U, Rennie L et al (2021) Dual-Task effects during a motor-cognitive Task in Parkinson’s Disease: patterns of prioritization and the influence of Cognitive Status. Neurorehabil Neural Repair 35:356–366. 10.1177/1545968321999053 33719728 10.1177/1545968321999053 PMC8073879

[CR12] Kopecek M, Stepankova H, Lukavsky J et al (2017) Montreal cognitive assessment (MoCA): normative data for old and very old Czech adults. Appl Neuropsychology: Adult 24:23–29. 10.1080/23279095.2015.1065261 10.1080/23279095.2015.1065261 27144665

[CR13] Litvan I, Goldman JG, Tröster AI et al (2012) Diagnostic criteria for mild cognitive impairment in Parkinson’s disease: Movement Disorder Society Task Force guidelines. Mov Disord 27(3):349–356. 10.1002/mds.24893 22275317 10.1002/mds.24893 PMC3641655

[CR14] Liu Y-C, Yang Y-R, Yeh N-C et al (2022) Multiarea Brain activation and gait deterioration during a cognitive and Motor Dual Task in individuals with Parkinson Disease. J Neurol Phys Ther 46:260–269. 10.1097/NPT.0000000000000402 35404916 10.1097/NPT.0000000000000402

[CR15] Morris M, Iansek R, Matyas T, Summers J (1998) Abnormalities in the stride length-cadence relation in parkinsonian gait. Mov Disord 13:61–69. 10.1002/mds.870130115 9452328 10.1002/mds.870130115

[CR16] Nieuwhof F, Bloem BR, Reelick MF et al (2017) Impaired dual tasking in Parkinson’s disease is associated with reduced focusing of cortico-striatal activity. Brain 140:1384–1398. 10.1093/brain/awx042 28335024 10.1093/brain/awx042

[CR17] Postuma RB, Berg D, Stern M et al (2015) MDS clinical diagnostic criteria for Parkinson’s disease: MDS-PD Clinical Diagnostic Criteria. Mov Disord 30:1591–1601. 10.1002/mds.26424 26474316 10.1002/mds.26424

[CR18] Raffegeau TE, Krehbiel LM, Kang N et al (2019) A meta-analysis: Parkinson’s disease and dual-task walking. Parkinsonism Relat Disord 62:28–35. 10.1016/j.parkreldis.2018.12.012 30594454 10.1016/j.parkreldis.2018.12.012 PMC8487457

[CR19] Tessitore A, Amboni M, Cirillo G et al (2012) Regional Gray Matter Atrophy in patients with Parkinson Disease and Freezing of Gait. AJNR Am J Neuroradiol 33:1804–1809. 10.3174/ajnr.A3066 22538070 10.3174/ajnr.A3066 PMC7964749

[CR20] The MathWorks Inc (2022) MATLAB version: 9.13.0 (R2022b)

[CR21] Varalta V, Picelli A, Fonte C et al (2015) Relationship between cognitive performance and motor dysfunction in patients with Parkinson’s Disease: a pilot cross-sectional study. Biomed Res Int 2015:1–6. 10.1155/2015/365959 10.1155/2015/365959 PMC4396143 25918713

[CR22] Wall JC, Bell C, Campbell S, Davis J (2000) The timed get-up-and-Go test revisited: measurement of the component tasks. J Rehabil Res Dev 37:109–113 10847578

[CR23] Watanabe H, Senda J, Kato S et al (2013) Cortical and subcortical brain atrophy in Parkinson’s disease with visual hallucination. Mov Disord 28:1732–1736. 10.1002/mds.25641 24150865 10.1002/mds.25641

[CR24] Wilson H, Niccolini F, Pellicano C, Politis M (2019) Cortical thinning across Parkinson’s disease stages and clinical correlates. J Neurol Sci 398:31–38. 10.1016/j.jns.2019.01.020 30682518 10.1016/j.jns.2019.01.020

[CR25] Zampieri C, Salarian A, Carlson-Kuhta P, Aminian K, Nutt JG, Horak FB (2010) The instrumented timed up and go test: potential outcome measure for disease modifying therapies in Parkinson’s disease. J Neurol Neurosurg Psychiatry 81(2):171–176. 10.1136/jnnp.2009.173740 19726406 10.1136/jnnp.2009.173740 PMC3065923

[CR26] Zanardi APJ, Da Silva ES, Costa RR et al (2021) Gait parameters of Parkinson’s disease compared with healthy controls: a systematic review and meta-analysis. Sci Rep 11:752. 10.1038/s41598-020-80768-2 33436993 10.1038/s41598-020-80768-2 PMC7804291

[CR27] Zhang X, Fan W, Yu H et al (2022) Single- and dual-task gait performance and their diagnostic value in early-stage Parkinson’s disease. Front Neurol 13:974985. 10.3389/fneur.2022.974985 36313494 10.3389/fneur.2022.974985 PMC9615249

