# Supplementary material for: Impaired dual-task gait in Parkinson’s disease is associated with brain morphology changes
Source: J Neural Transm (Vienna). 2024 Feb 28;131(12):1389–95. doi: 10.1007/s00702-024-02758-2 (PMC11608385; doi:10.1007/s00702-024-02758-2)
Supplement: Supplementary file 1 — Supplementary Material 1 [file 702_2024_2758_MOESM1_ESM.docx]

Supplementary Table 1:

Differences in single- and dual-task gait

|  | HC  mean (sd) | PD  mean (sd) | p-value | PD-nDTC  mean (sd) | PD-iDTC  mean (sd) | p-value |
| --- | --- | --- | --- | --- | --- | --- |
| Speed [cm/s] | 121 (13.6) | 111 (16.8) | **< 0.001** | 113 (16.9) | 106 (15.9) | 0.12 |
| Cadence [steps/min] | 111 (7.57) | 109 (9.67) | 0.089 | 110 (9.91) | 105 (8.68) | 0.11 |
| Stride length [cm] | 130 (10.1) | 122 (12.3) | **< 0.001** | 123 (12.1) | 120 (13.0) | 0.39 |
| Speed cost | -0.055 (0.076) | -0.105 (0.103) | **0.007** | - | - | - |
| Cadence cost | -0.026 (0.041) | -0.050 (0.066) | **0.029** | - | - | - |
| Stride length cost | -0.033 (0.050) | -0.061 (0.065) | **0.014** | - | - | - |
| PCA – first component | -0.064 (0.089) | -0.120 (0.119) | **0.007** | - | - | - |

Abbreviations: HC, Healthy controls; PD, Parkinson disease; PD-nDTC, PD patients with normal dual-task, PD-iDTC - with abnormally increased DTC
